# Psychometric validation of a digital literacy scale for physical education teachers in China

**DOI:** 10.3389/fpsyg.2026.1871523

**Published:** 2026-07-20

**Authors:** Jiaheng Wang, Xiaolin Zhang, Qingju Shao, Xuefang Li, Yi Yang, Jingtao Wu, Feng Xue

**Affiliations:** 1 School of Physical Education, Sichuan Normal University, Chengdu, China; 2 School of Marxism, Chongqing Normal University, Chongqing, China; 3 School of Humanities and Law, Chengdu University of Technology, Chengdu, China; 4 School of Physical Education, Leshan Normal University, Leshan, China

**Keywords:** digital literacy, educational psychology, physical education teachers, psychometric validation, scale development, teacher digital competence, technology-supported teaching

## Abstract

**Introduction:**

Teacher digital literacy is increasingly important in technology-supported education, but subject-specific measurement tools for physical education remain limited. This study aimed to develop and psychometrically validate a digital literacy scale for physical education teachers in China.

**Methods:**

A cross-sectional survey was conducted with 836 physical education teachers. Guided by educator digital competence frameworks and movement-oriented teaching characteristics, an initial 25-item instrument was developed. Item analysis, reliability testing, exploratory factor analysis, confirmatory factor analysis, convergent validity analysis, and one-way analysis of variance were used to examine item quality, factor structure, reliability, construct validity, and differences across school levels.

**Results:**

The analyses supported a final 23-item, five-factor structure covering digital instructional design and implementation, digital resource development and integration, digital assessment and feedback, digital interaction and student empowerment, and digital professional development. Cronbach’s alpha coefficients ranged from 0.923 to 0.968. Exploratory factor analysis explained 85.554% of the total variance. Confirmatory factor analysis showed acceptable model fit, with CFI = 0.958, TLI = 0.952, and RMSEA = 0.078. Composite reliability and average variance extracted values also supported convergent validity. No significant differences were found across school levels.

**Discussion:**

The scale provides a reliable and valid subject-specific tool for assessing physical education teachers’ perceived digital literacy. It may support future research, teacher education, needs assessment, and professional development in technology-supported physical education.

## 1 Introduction

Digital transformation has reshaped how teaching is organized, delivered, and evaluated. In psychology and education research, teacher digital literacy is increasingly treated as a multidimensional professional competence rather than a narrow set of technical skills. International frameworks such as DigCompEdu describe educators’ digital competence in relation to professional engagement, digital resources, teaching and learning, assessment, learner empowerment, and the facilitation of learners’ own digital competence (Redecker and Punie, 2017). The UNESCO ICT Competency Framework for Teachers similarly emphasizes that digital technologies should be integrated with pedagogy, curriculum, assessment, and professional development (UNESCO, 2018). The OECD has also described teacher digital competence as a necessary component of effective digital education ecosystems (OECD, 2023). Conceptual perspectives such as technological pedagogical content knowledge and the teacher digital competency framework further suggest that meaningful technology use depends on the integration of technology, pedagogy, subject matter, and reflective professional judgment (Mishra and Koehler, 2006; Falloon, 2020).

Despite the rapid growth of research on teacher digital competence, consistent measurement remains challenging. Recent reviews have shown that definitions, dimensions, and operational indicators differ considerably across studies (Smestad et al., 2023; Claro et al., 2024). Comparative analyses of teacher digital competence frameworks show broad convergence around teaching, assessment, communication, digital resources, and professional learning, while also demonstrating that the practical meaning of these domains depends on disciplinary and institutional contexts (Velandia Rodriguez et al., 2022). Several validated instruments have been developed for general education settings, including multidimensional scales of teachers’ digital competence and structured tools for assessing educators’ digital competence (Alarcón et al., 2020; Gümüş and Kukul, 2023; Ergül and Taşar, 2023; Aydin et al., 2024). These studies provide important foundations for psychometric research on digital competence, but they do not fully address how digital literacy is enacted in discipline-specific teaching practices.

Physical education is a particularly important context for subject-specific measurement because learning is cognitive, social, embodied, and performance-based. In physical education, digital technologies are used not only for presentation, communication, or platform management but also for movement demonstration, video capture, wearable monitoring, performance analysis, activity tracking, and feedback on bodily performance (Casey et al., 2017; Wallace et al., 2023). A critical review of technology-enhanced learning in physical education showed that pedagogical value depends on how digital tools are embedded in movement-oriented learning tasks (Sargent and Calderón, 2022). Empirical studies have also reported that physical education teachers’ perceived digital competence is uneven across teaching functions and school contexts (Rojo-Ramos et al., 2020; Martínez-Rico et al., 2022). At the same time, physical education teachers frequently report barriers such as limited resources, insufficient professional development, school-level constraints, and uncertainty about how digital tools fit bodily and performance-based learning (Saiz-González et al., 2025).

These issues create a psychometric and practical problem. General teacher digital competence scales may capture broad technology use, but they may overlook the ways digital tools are used to support movement learning, physical activity engagement, performance feedback, and data-informed instruction in physical education. Without a subject-specific instrument, researchers and teacher educators have limited evidence for diagnosing physical education teachers’ professional development needs, evaluating technology-related training, and examining how digital competence relates to instructional practice. Scale development literature also emphasizes that valid measurement requires a clear construct definition, transparent item development, and evidence that items align with the intended domain (Boateng et al., 2018; DeVellis and Thorpe, 2021). Recent calls for context-sensitive research on digital technology in physical education and physical education teacher education therefore support the development of a discipline-specific measurement tool (Østerlie et al., 2022; Østerlie et al., 2025).

The present study developed and psychometrically validated a digital literacy scale for physical education teachers in China. The study was guided by a pre-specified Chinese Physical Education Digital Literacy framework informed by DigCompEdu and adapted to the pedagogical realities of movement-oriented instruction (Redecker and Punie, 2017; Sullivan et al., 2024). Rather than treating digital literacy as a single general ability, the framework operationalized five dimensions: digital instructional design and implementation, digital resource development and integration, digital assessment and feedback, digital interaction and student empowerment, and digital professional development. In addition to testing the factor structure and reliability of the instrument, the study examined whether scores differed across school levels. This comparison was necessary because digital infrastructure, curricular tasks, student age, assessment expectations, and professional development opportunities may vary between primary schools, secondary schools, high schools, vocational colleges, and universities. If school-level differences were present, they would have implications for differentiated teacher training and scale interpretation; if they were absent, this would suggest that other contextual factors may be more important than school level in explaining self-reported digital literacy. The study addressed three research questions: (1) What factor structure underlies physical education teachers’ digital literacy? (2) Does the proposed scale demonstrate acceptable reliability and construct validity? (3) Do physical education teachers’ digital literacy scores differ across school levels?

## 2 Materials and methods

### 2.1 Study design and participants

This study used a cross-sectional survey design and psychometric validation approach. A total of 836 valid questionnaires from physical education teachers were included in the final analysis. The sample covered urban primary schools, urban secondary schools, urban high schools, rural or township primary schools, rural or township secondary schools, rural or township high schools, higher vocational colleges, and undergraduate institutions. This sampling scope allowed the study to examine the proposed scale across a broad range of educational settings.

Of the respondents, 586 were men (70.1%) and 250 were women (29.9%). Regarding age, 19.50% were under 30 years, 35.41% were 31–40 years, 30.02% were 41–50 years, and 15.07% were 51 years or above. Teaching experience was also widely distributed: 20.22% had 1–5 years, 16.63% had 6–10 years, 26.56% had 11–20 years, and 36.60% had more than 21 years of experience. Detailed participant characteristics are shown in Table 1.

**Table 1:** Operational definitions of the five domains and links to item wording.

**Domain**	**Operational definition**	**Item wording and PE-specific expression**
Digital instructional design and implementation	Teachers’ perceived ability to design and enact physical education lessons using digital tools in ways that support movement-oriented instruction.	Items B1-B5 refer to digital lesson planning, digital devices in PE classes, differentiated digital content, digital task assignment and tracking, and wearable-device-supported adjustment of teaching.
Digital resource development and integration	Teachers’ perceived ability to locate, produce, integrate, and share digital resources for physical education teaching.	Items C1-C5 refer to searching resources on educational platforms, producing PE videos or animations, integrating videos/images/data, developing fitness test forms or exercise cards, and sharing resources online.
Digital assessment and feedback	Teachers’ perceived ability to use digital information to represent students’ motor performance and provide feedback that informs subsequent instruction.	Initial items D1-D5 covered digital assessment tools, data analysis, visualized performance trends, personalized digital feedback, and data-informed instructional adjustment. The retained items D3-D5 represent the feedback cycle more coherently.
Digital interaction and student empowerment	Teachers’ perceived ability to use digital tools to support communication, autonomous exercise, motivation, and student engagement in physical activity.	Items F1-F5 refer to online interaction, guiding students to use exercise apps, designing digital participation tasks, using digital resources to improve motor skills, and organizing online challenges.
Digital professional development	Teachers’ perceived ability to use digital platforms and tools for continued learning, reflection, peer exchange, and policy engagement.	Items G1-G5 refer to learning emerging digital tools, participating in online professional development, using reflection tools, joining professional communities, and following digital education policies or programs.

PE = physical education. The full item wording is provided in Table A1.

### 2.2 Instrument development and preliminary evaluation

The questionnaire was developed under the Chinese Physical Education Digital Literacy framework. The framework was conceptually aligned with DigCompEdu and related scholarship on digital pedagogy in physical education, but it translated broad competence domains into subject-specific practices, including movement demonstration, task tracking, digitally enabled assessment, performance feedback, learner engagement, and professional learning (Redecker and Punie, 2017; Wallace et al., 2023; Sullivan et al., 2024). To make the construct definitions transparent, Table 1 provides the operational definition of each domain and explains how each definition was reflected in the item wording.

Before the main survey, the questionnaire underwent a two-round preliminary evaluation and expert-informed review. Both rounds were conducted before formal data collection and involved the same group of approximately 15–20 physical education teachers and experts from physical education, education, and related fields. The two rounds were separated by an interval of more than 15 days. Through group discussion, the participants reviewed the questionnaire content, item wording, domain relevance, and contextual appropriateness. Based on their feedback, the research team revised the questionnaire content and item wording to improve readability, domain alignment, and relevance to physical education teaching practice. The participants in the preliminary evaluation were not included in the final sample of 836 teachers. This preliminary process helped strengthen the content relevance and contextual appropriateness of the item pool before large-scale psychometric validation.

The initial instrument contained two parts. The first part collected demographic information, including gender, age, teaching experience, educational attainment, school level, weekly teaching load, and commonly used digital teaching tools. The second part contained 25 substantive items distributed across five dimensions. Each dimension initially included five items. All substantive items were rated on a five-point Likert-type scale ranging from 1 to 5, with higher scores indicating stronger self-reported digital literacy. The full item wording is provided in Table A1.

### 2.3 Data collection and ethical considerations

Data were collected through Wenjuanxing, an online questionnaire platform, after ethics approval was obtained and during the approved study period from November 20, 2024 to December 31, 2024. Participants accessed the questionnaire through an electronic link or QR code and completed it anonymously. Before completing the questionnaire, participants were informed of the purpose of the study, the anonymous processing of responses, the voluntary nature of participation, and their right to withdraw. Electronic informed consent was obtained before participants began the questionnaire. All core questionnaire items were set as required fields. Before analysis, the exported dataset was screened for eligibility, completeness, and potential invalid responses. Eligibility was determined by whether the respondent was a physical education teacher or a relevant teaching professional. Potential duplicate or invalid responses were checked by considering IP information, demographic consistency, and response patterns. After data screening, 836 valid responses were retained for analysis. The study was approved by the Ethics Committee of the Academic Committee of Leshan Normal University (approval no. LSNU: 103324-RO).

### 2.4 Statistical analysis

The analysis proceeded in several steps. First, descriptive statistics, skewness, kurtosis, coefficients of variation, and corrected item-total correlations were calculated to examine item quality. Second, Cronbach’s alpha coefficients were used to examine internal consistency. Third, exploratory factor analysis was conducted to examine the latent structure of the proposed scale. The Kaiser-Meyer-Olkin index and Bartlett’s test of sphericity were used to evaluate whether the data were suitable for factor analysis. Principal component extraction with varimax rotation was used to obtain the main loading pattern. Items were interpreted by considering factor loadings, cross-loadings, theoretical coherence, and the clarity of the retained domain structure. Fourth, confirmatory factor analysis was used to verify the retained measurement structure and to estimate standardized factor loadings, composite reliability, and average variance extracted. Model fit was interpreted using commonly applied criteria in structural equation modeling: CFI and TLI values of 0.90 or above were treated as indicating acceptable fit, values near 0.95 as indicating good fit, and RMSEA values below 0.08 as indicating acceptable fit; the chi-square divided by degrees of freedom ratio was also considered as a descriptive index of fit (Hu and Bentler, 1999; Kline, 2016; Hair et al., 2019). Composite reliability values above 0.70 and average variance extracted values around or above 0.50 were interpreted as supporting convergent validity (Fornell and Larcker, 1981; Hair et al., 2019). Fifth, one-way analysis of variance was performed to compare mean scores across school levels, and eta squared was reported as an effect-size indicator for school-level differences. Statistical significance was judged at *p* < 0.05.

## 3 Results

### 3.1 Participant characteristics

The sample represented a wide range of demographic and institutional backgrounds (Table 1). Urban primary school teachers accounted for the largest proportion of the sample (32.78%), followed by urban secondary school teachers (18.18%). In terms of weekly teaching load, most respondents reported teaching 11–15 lessons per week (42.46%) or 16–20 lessons per week (32.54%).

### 3.2 Item descriptive statistics

The descriptive statistics for the 25 observed items are shown in Table 2. The means of all items ranged from 3.481 to 4.008, indicating that physical education teachers’ self-assessed digital literacy was generally at a moderately high level. Item B5, which focused on integrating wearable devices to monitor students’ physical performance and adjust teaching plans, had the lowest mean (*M* = 3.481, SD = 1.234). Item C1, which focused on searching for and downloading digital resources suitable for physical education teaching, had the highest mean (*M* = 4.008, SD = 0.989).

**Table 2:** Demographic characteristics of participants.

**Variable**	**Category**	***n***	**%**	**Cumulative %**
Gender	Male	586	70.1	70.1
Gender	Female	250	29.9	100
Age	Under 30 years	163	19.50	19.50
Age	31–40 years	296	35.41	54.90
Age	41–50 years	251	30.02	84.93
Age	51 years or above	126	15.07	100
Teaching experience	1–5 years	169	20.22	20.22
Teaching experience	6–10 years	139	16.63	36.84
Teaching experience	11–20 years	222	26.56	63.40
Teaching experience	More than 21 years	306	36.60	100
Educational attainment	Junior college or below	52	6.22	6.22
Educational attainment	Bachelor’s degree	627	75.00	81.22
Educational attainment	Master’s degree	124	14.83	96.05
Educational attainment	Doctoral degree	33	3.95	100
School level	Urban primary school	274	32.78	32.78
School level	Urban secondary school	152	18.18	50.96
School level	Urban high school	86	10.29	61.24
School level	Rural/township primary school	90	10.77	72.01
School level	Rural/township secondary school	95	11.36	83.37
School level	Rural/township high school	27	3.23	86.60
School level	Higher vocational college	26	3.11	89.71
School level	Undergraduate institution	86	10.29	100
Weekly PE lessons	1–5 lessons	44	5.26	5.26
Weekly PE lessons	6–10 lessons	137	16.39	21.65
Weekly PE lessons	11–15 lessons	355	42.46	64.11
Weekly PE lessons	16–20 lessons	272	32.54	96.65
Weekly PE lessons	More than 21 lessons	28	3.35	100
Total	Total	836	100	100

Percentages may not sum exactly because of rounding.

Overall, items related to basic resource use and routine digital instructional support were rated more positively than items requiring advanced monitoring or analytics. This pattern is consistent with prior studies showing that physical education teachers often feel more comfortable with accessible instructional technologies than with data-intensive or equipment-dependent practices (Wallace et al., 2023; Saiz-González et al., 2025).

### 3.3 Reliability evidence

The internal consistency results are reported in Table 3. Corrected item-total correlations ranged from 0.697 to 0.928, indicating that the items contributed meaningfully to their intended dimensions. Cronbach’s alpha coefficients were 0.923 for digital instructional design and implementation, 0.946 for digital resource development and integration, 0.962 for digital assessment and feedback, 0.968 for digital interaction and student empowerment, and 0.960 for digital professional development. These results indicated strong internal coherence for the five proposed dimensions (Table 4).

**Table 3:** Descriptive statistics of the observed items.

**Item**	**Mean ± SD**	**Variance**	**95% CI (LL)**	**95% CI (UL)**	**Kurtosis**	**Skewness**	**CV**
B1	3.901 ± 1.069	1.143	3.828	3.973	0.822	−1.109	27.413%
B2	3.822 ± 1.087	1.181	3.748	3.895	0.602	−1.044	28.440%
B3	3.751 ± 1.077	1.160	3.678	3.824	0.414	−0.935	28.707%
B4	3.840 ± 1.092	1.193	3.766	3.914	0.455	−1.016	28.451%
B5	3.481 ± 1.234	1.522	3.397	3.564	−0.518	−0.660	35.440%
C1	4.008 ± 0.989	0.978	3.941	4.075	1.478	−1.252	24.677%
C2	3.682 ± 1.066	1.137	3.610	3.754	0.039	−0.747	28.961%
C3	3.900 ± 0.986	0.972	3.833	3.966	1.143	−1.091	25.282%
C4	3.715 ± 1.039	1.081	3.645	3.786	0.268	−0.815	27.978%
C5	3.813 ± 0.993	0.985	3.746	3.881	0.716	−0.937	26.032%
D1	3.823 ± 0.994	0.989	3.756	3.890	0.606	−0.943	26.013%
D2	3.873 ± 1.000	0.999	3.805	3.941	0.833	−1.041	25.812%
D3	3.705 ± 1.066	1.135	3.632	3.777	0.214	−0.837	28.763%
D4	3.706 ± 1.047	1.097	3.635	3.777	0.245	−0.839	28.258%
D5	3.791 ± 1.001	1.002	3.723	3.859	0.737	−0.987	26.402%
F1	3.774 ± 1.019	1.037	3.705	3.843	0.399	−0.872	26.989%
F2	3.780 ± 1.015	1.029	3.711	3.849	0.339	−0.859	26.841%
F3	3.740 ± 1.044	1.091	3.670	3.811	0.278	−0.844	27.920%
F4	3.780 ± 1.007	1.015	3.712	3.848	0.568	−0.922	26.653%
F5	3.769 ± 1.029	1.059	3.699	3.839	0.362	−0.861	27.305%
G1	3.706 ± 1.036	1.073	3.636	3.776	0.349	−0.841	27.947%
G2	3.782 ± 0.991	0.982	3.715	3.849	0.818	−0.981	26.206%
G3	3.831 ± 0.966	0.933	3.766	3.897	0.861	−0.968	25.214%
G4	3.910 ± 0.958	0.918	3.845	3.975	1.282	−1.123	24.498%
G5	3.858 ± 0.967	0.934	3.792	3.923	0.776	−0.957	25.055%

B = digital instructional design and implementation; C = digital resource development and integration; D = digital assessment and feedback; F = digital interaction and student empowerment; G = digital professional development; CV = coefficient of variation.

**Table 4:** Reliability analysis of the observed items.

**Item**	**Corrected item-total correlation**	**Alpha if item deleted**	**Cronbach’s alpha**
B1	0.797	0.906	0.923
B2	0.860	0.894	0.923
B3	0.846	0.897	0.923
B4	0.818	0.902	0.923
B5	0.697	0.929	0.923
C1	0.818	0.939	0.946
C2	0.844	0.935	0.946
C3	0.904	0.924	0.946
C4	0.841	0.935	0.946
C5	0.855	0.933	0.946
D1	0.862	0.957	0.962
D2	0.841	0.961	0.962
D3	0.908	0.950	0.962
D4	0.921	0.948	0.962
D5	0.928	0.947	0.962
F1	0.873	0.967	0.968
F2	0.925	0.958	0.968
F3	0.912	0.960	0.968
F4	0.927	0.958	0.968
F5	0.909	0.961	0.968
G1	0.866	0.955	0.960
G2	0.916	0.946	0.960
G3	0.888	0.950	0.960
G4	0.883	0.951	0.960
G5	0.887	0.951	0.960

CITC = corrected item-total correlation.

### 3.4 Exploratory factor analysis

The data were suitable for factor analysis. The Kaiser-Meyer-Olkin index was 0.980, and Bartlett’s test of sphericity was significant (χ2 = 28760.231, df = 300, *p* < 0.001). The exploratory factor analysis results are reported in Table 5. A five-factor solution was retained. The rotated eigenvalues of the five factors were 6.154, 4.842, 4.255, 3.463, and 2.674, respectively. The corresponding variance contributions were 24.616, 19.367, 17.021, 13.852, and 10.698%, and the cumulative explained variance reached 85.554%. These results indicated that the five-factor structure explained a large proportion of total variance.

**Table 5:** Exploratory factor analysis results.

**Item**	**Factor 1**	**Factor 2**	**Factor 3**	**Factor 4**	**Factor 5**	**Communality**
B1		0.790				0.831
B2		0.771				0.855
B3		0.694				0.818
B4		0.707				0.790
B5		0.479				0.840
C1				0.465		0.843
C2				0.667		0.843
C3				0.620		0.893
C4				0.641		0.841
C5				0.560		0.818
D3					0.571	0.898
D4					0.567	0.902
D5					0.464	0.880
F1			0.615			0.847
F2			0.695			0.913
F3			0.669			0.900
F4			0.670			0.908
F5			0.661			0.889
G1	0.691					0.817
G2	0.721					0.880
G3	0.690					0.854
G4	0.730					0.868
G5	0.734					0.857
Rotated eigenvalue	6.154	4.842	4.255	3.463	2.674	-
Rotated variance explained (%)	24.616	19.367	17.021	13.852	10.698	-
Cumulative variance explained (%)	24.616	43.982	61.004	74.856	85.554	-

The table reports the main retained loading pattern and item communalities. D1 and D2 were evaluated in the initial 25-item solution but were not retained in the final validation model because they did not cluster cleanly with D3-D5 and showed weaker domain specificity.

The loading pattern was broadly consistent with the conceptual framework. Items B1-B5 loaded on digital instructional design and implementation, items C1-C5 loaded on digital resource development and integration, items F1-F5 loaded on digital interaction and student empowerment, and items G1-G5 loaded on digital professional development. For the digital assessment and feedback dimension, items D3-D5 formed the retained factor in the final validation structure. The decision not to retain D1 and D2 was supported by both theoretical and statistical considerations. Theoretically, D1 and D2 reflected general digital assessment awareness and generic data-analysis practices, whereas D3–D5 captured a more coherent physical education feedback cycle: visualizing performance change, providing personalized feedback, and adjusting instruction based on assessment data. Statistically, in the five-factor rotated solution including the initial 25 items, D1 and D2 did not cluster cleanly with D3–D5. Their highest loadings were on a factor that was not the retained digital assessment and feedback factor, and both items also showed substantial cross-loadings across several factors. By contrast, D3–D5 formed a more interpretable and parsimonious factor representing visualized performance feedback and data-informed instructional adjustment. Therefore, the final validation model retained the 23-item structure.

### 3.5 Confirmatory factor analysis and convergent validity

**Figure 1:**
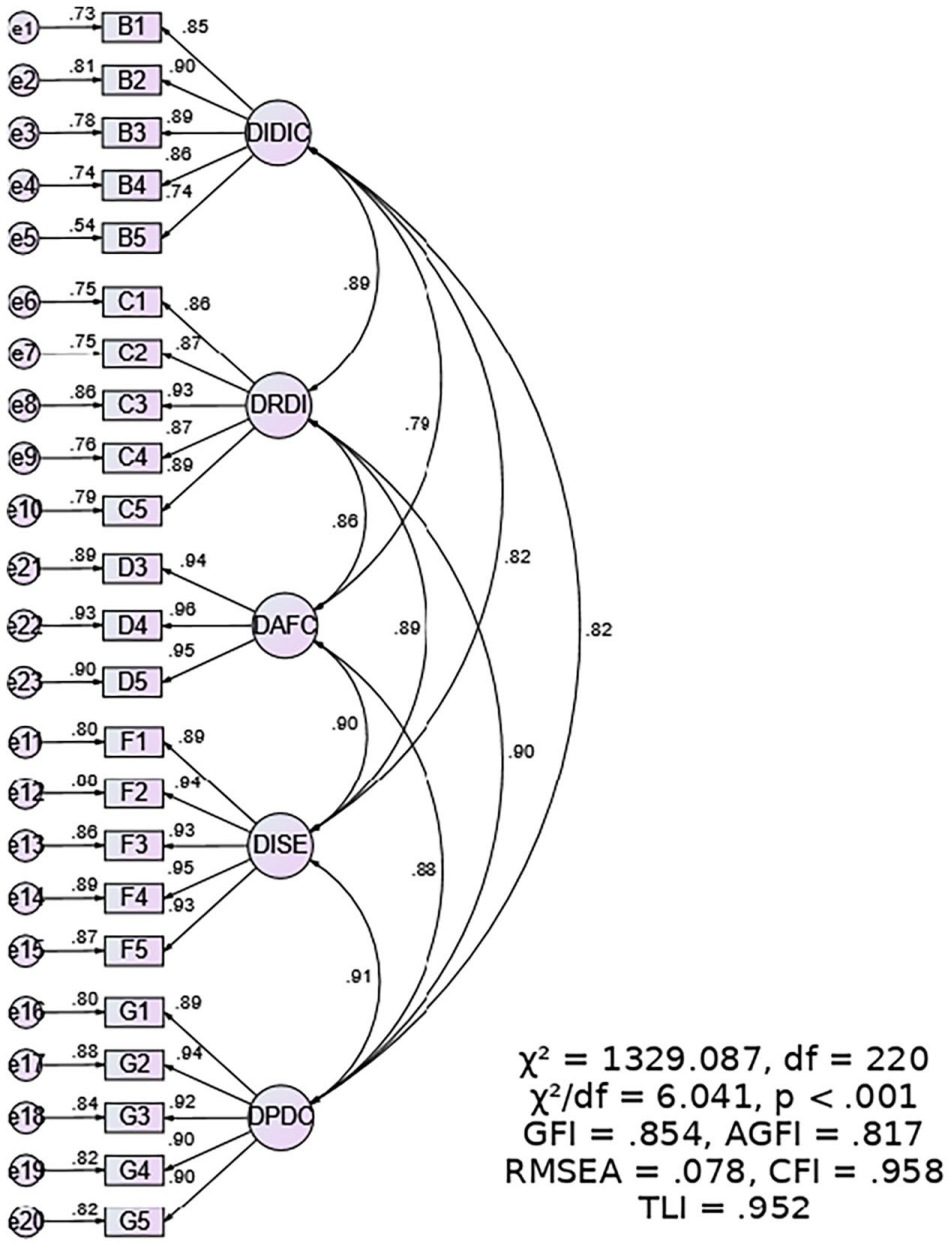
Confirmatory factor model of the PE teachers’ digital literacy scale. DIDIC = digital instructional design and implementation competence; DRDI = digital resource development and integration competence; DAFC = digital assessment and feedback competence; DISE = digital interaction and student empowerment competence; DPDC = digital professional development competence.

The confirmatory factor model is presented in Figure 1, and the path coefficients are shown in Table 6. The model fit indices indicated acceptable fit: χ2 = 1329.087, df = 220, χ2 / df = 6.041, GFI = 0.854, AGFI = 0.817, RMSEA = 0.078, CFI = 0.958, and TLI = 0.952. Although the χ2 / df ratio was higher than a strict cutoff, this index is sensitive to large samples. The incremental fit indices and RMSEA supported an acceptable retained measurement model according to commonly used criteria (Hu and Bentler, 1999; Kline, 2016; Hair et al., 2019). Standardized factor loadings ranged from 0.735 to 0.963.

**Table 6:** CFA path coefficients, composite reliability, and average variance extracted.

**Item**	**Latent variable**	**Unstandardized**	**Standardized**	**S.E.**	**C.R.**	**Composite reliability**	**AVE**
B1	DIDIC	1.000	0.852			0.927	0.721
B2	DIDIC	1.072	0.899	0.030	35.302	0.927	0.721
B3	DIDIC	1.046	0.885	0.030	34.322	0.927	0.721
B4	DIDIC	1.032	0.861	0.032	32.607	0.927	0.721
B5	DIDIC	0.995	0.735	0.039	25.238	0.927	0.721
C1	DRDI	1.000	0.864			0.947	0.781
C2	DRDI	1.082	0.868	0.031	34.506	0.947	0.781
C3	DRDI	1.069	0.926	0.027	39.568	0.947	0.781
C4	DRDI	1.061	0.872	0.030	34.888	0.947	0.781
C5	DRDI	1.031	0.888	0.029	36.154	0.947	0.781
D3	DAFC	1.000	0.943			0.966	0.904
D4	DAFC	1.003	0.963	0.016	61.878	0.966	0.904
D5	DAFC	0.943	0.947	0.016	57.478	0.966	0.904
F1	DISE	1.000	0.893			0.969	0.861
F2	DISE	1.046	0.938	0.023	45.563	0.969	0.861
F3	DISE	1.067	0.930	0.024	44.397	0.969	0.861
F4	DISE	1.047	0.946	0.022	46.663	0.969	0.861
F5	DISE	1.055	0.933	0.024	44.807	0.969	0.861
G1	DPDC	1.000	0.893			0.960	0.829
G2	DPDC	1.004	0.936	0.022	44.828	0.960	0.829
G3	DPDC	0.957	0.916	0.023	42.274	0.960	0.829
G4	DPDC	0.936	0.903	0.023	40.808	0.960	0.829
G5	DPDC	0.946	0.905	0.023	40.964	0.960	0.829

AVE = average variance extracted.

Convergent validity was also satisfactory. Composite reliability values ranged from 0.927 to 0.969, and average variance extracted values ranged from 0.721 to 0.904. Specifically, digital instructional design and implementation yielded composite reliability = 0.927 and average variance extracted = 0.721; digital resource development and integration yielded composite reliability = 0.947 and average variance extracted = 0.781; digital assessment and feedback yielded composite reliability = 0.966 and average variance extracted = 0.904; digital interaction and student empowerment yielded composite reliability = 0.969 and average variance extracted = 0.861; and digital professional development yielded composite reliability = 0.960 and average variance extracted = 0.829. These findings supported the adequacy of the retained indicators.

### 3.6 Differences across school levels

The one-way analysis of variance results are shown in Table 7. No statistically significant differences were observed across school levels for digital instructional design and implementation, digital resource development and integration, digital assessment and feedback, digital interaction and student empowerment, or digital professional development. The effect sizes were also small (η2 values ranging from 0.010 to 0.014), indicating that school level explained only a small proportion of variance in self-reported digital literacy scores. Although mean values varied slightly across school levels, the overall pattern suggested that the sampled physical education teachers shared relatively similar self-reported digital literacy profiles across the five dimensions.

**Table 7:** One-way ANOVA of digital literacy across school levels.

**Dimension**	**Urban primary**	**Urban secondary**	**Urban high**	**Rural primary**	**Rural secondary**	**Rural high**	**Higher vocational**	**Undergraduate**	***F***	***p***	**η** ^**2**^
DIDIC	3.85 ± 1.01	3.78 ± 0.98	3.76 ± 1.05	3.69 ± 0.90	3.52 ± 0.86	3.59 ± 0.83	3.65 ± 1.19	3.84 ± 0.91	1.523	0.156	0.013
DRDI	3.88 ± 0.93	3.74 ± 0.93	3.78 ± 0.99	3.65 ± 0.91	3.66 ± 0.75	3.57 ± 0.77	3.56 ± 1.17	3.80 ± 0.85	1.397	0.203	0.012
DAFC	3.85 ± 1.03	3.73 ± 0.98	3.77 ± 1.01	3.57 ± 1.06	3.63 ± 0.91	3.72 ± 0.79	3.50 ± 1.19	3.70 ± 0.99	1.281	0.256	0.011
DISE	3.90 ± 0.97	3.69 ± 0.98	3.87 ± 0.99	3.63 ± 0.97	3.64 ± 0.86	3.74 ± 0.82	3.61 ± 1.23	3.73 ± 0.90	1.661	0.115	0.014
DPDC	3.93 ± 0.93	3.74 ± 0.94	3.88 ± 0.92	3.77 ± 0.89	3.74 ± 0.79	3.73 ± 0.70	3.62 ± 1.15	3.77 ± 0.92	1.151	0.329	0.010

Values for school-level groups are M ± SD. η^2^ = eta squared effect size.

## 4 Discussion

### 4.1 Principal findings

This study developed and psychometrically validated a subject-specific digital literacy scale for physical education teachers in China. The findings supported a five-dimensional understanding of physical education teachers’ digital literacy and provided evidence that the instrument had acceptable psychometric quality. The retained dimensions were conceptually aligned with major educator digital competence frameworks while also reflecting physical education-specific practices, including movement-oriented lesson design, digital resource integration, performance feedback, student engagement through digital tasks, and professional learning.

The results also showed that teachers reported stronger competence in locating and integrating digital resources than in using wearable devices and more advanced assessment-related technologies. This pattern is meaningful because it suggests that digital literacy in physical education may develop unevenly across functions. Low-threshold practices such as searching for resources and using digital teaching materials may be easier to adopt, whereas data-intensive practices such as wearable monitoring and digital assessment require stronger infrastructure, training, and pedagogical interpretation. This interpretation is consistent with previous evidence that physical education teachers’ digital competence is shaped by access, support, training, and perceived pedagogical fit rather than by technology availability alone (Rojo-Ramos et al., 2020; Martínez-Rico et al., 2022; Saiz-González et al., 2025).

### 4.2 Psychometric and theoretical contribution

The study contributes to psychology and measurement research by extending teacher digital competence measurement into a subject-specific and embodied learning context. Scale development research emphasizes the importance of defining the target construct, aligning items with construct domains, and gathering multiple forms of validity evidence (Boateng et al., 2018; DeVellis and Thorpe, 2021). The present instrument follows this logic by translating general digital competence domains into observable physical education teaching practices. In doing so, it provides a measurement tool for examining how a professional competence construct is enacted in a discipline where learning is cognitive, embodied, performative, and context-dependent.

The five retained dimensions also show how broad frameworks such as DigCompEdu can be operationalized in a subject-specific way. Relative to general teacher digital competence instruments, the present scale highlights practices that are particularly relevant to physical education, such as digital lesson design for movement learning, visualized performance feedback, application-supported autonomous exercise, and technology-mediated participation tasks. This contribution is important because teacher competence is shaped not only by general digital skills but also by the psychological, pedagogical, and contextual demands of specific teaching domains (Falloon, 2020; Wallace et al., 2023; Sullivan et al., 2024).

### 4.3 Interpretation of the digital assessment and feedback domain

The refinement of the digital assessment and feedback domain deserves attention. D1 and D2 were initially designed to capture the use of digital assessment tools and data-analysis tools. However, the EFA evidence suggested that these items did not form as clear a factor with D3-D5 as expected. This pattern is theoretically plausible. General awareness of digital assessment tools and generic data analysis may represent broader digital tool familiarity, whereas visualized performance feedback and data-informed adjustment are closer to the actual feedback cycle in physical education. The retained D3-D5 items therefore represent a more coherent operationalization of assessment-related digital literacy in movement-oriented teaching. Nevertheless, the three-item structure also indicates that this domain should be refined in future versions of the scale by adding additional items on digital ethics, data protection, formative assessment, and responsible use of performance data.

### 4.4 Implications for teacher education and professional development

For practice, the scale can support teacher education programs, schools, and professional development providers in identifying physical education teachers’ strengths and weaknesses in technology-supported teaching. The relatively lower score for wearable-device integration suggests that professional development should move beyond basic digital resource use and address data-informed feedback, movement analysis, and pedagogically meaningful integration of digital monitoring tools. Teacher education programs may also use the scale to evaluate whether coursework and field experiences prepare future physical education teachers to design, implement, and assess digital learning activities in movement-based settings.

The non-significant differences across school levels respond to RQ3. One interpretation is that digital teaching expectations have become more widely shared across educational settings under the broader momentum of educational digitalization in China (Ministry of Education of the People’s Republic of China, 2022). Another possibility is that self-reported digital literacy is shaped more strongly by access, local infrastructure, professional development opportunities, and school support than by school level itself. This interpretation is consistent with international policy analyses emphasizing the importance of ecosystem-level support for teacher digital competence development (OECD, 2023). Therefore, future research should examine more specific contextual variables, such as access to devices, digital training experience, school technology policies, and frequency of digital tool use, rather than relying only on broad school-level categories.

### 4.5 Limitations and future research

Several limitations should be acknowledged. First, the data were self-reported, which may have inflated competence estimates. Future research should combine self-report measures with observational evidence, performance tasks, or external indicators of technology-supported teaching. Second, the study used a cross-sectional design, so the temporal stability of the scale was not tested. Future studies should examine test–retest reliability and longitudinal sensitivity. Third, although the questionnaire was refined through a two-round expert-informed preliminary evaluation, future studies may further strengthen the scale development process by using more formalized expert rating procedures and additional independent pilot samples before large-scale validation. Fourth, the digital assessment and feedback dimension retained only three indicators in the final validation model, indicating that this domain still requires refinement. Future revisions may consider adding indicators related to digital ethics, data protection, learner digital competence, and algorithmic or artificial intelligence-supported assessment in physical education. Fifth, future research should use independent calibration and validation samples, examine measurement invariance across gender, region, school level, and teaching experience, and incorporate external criterion variables such as teachers’ technology use frequency, school digital infrastructure, or student learning outcomes. Such work would help determine whether the scale can support cross-context comparison and program evaluation.

## 5 Conclusion

This study developed and psychometrically validated a digital literacy scale for physical education teachers in China. The retained five-dimensional structure captured key aspects of subject-specific digital literacy in physical education: digital instructional design and implementation, digital resource development and integration, digital assessment and feedback, digital interaction and student empowerment, and digital professional development. The scale demonstrated strong internal consistency and acceptable construct validity, supporting its use as a research and diagnostic tool. In relation to RQ3, no significant differences were found across school levels, and the effect sizes were small. This finding suggests that broad school-level categories may not be the primary source of variation in physical education teachers’ self-reported digital literacy; future studies should therefore examine more specific contextual factors such as infrastructure, training, school support, and technology-use experience. Further validation work should test the scale in broader samples, examine measurement invariance, and extend the framework to include digital ethics, safety, and learner digital competence more explicitly.

## Data Availability

The anonymized data supporting the conclusions of this article will be made available by the corresponding author upon reasonable request, subject to institutional data-protection requirements.

## Appendix

**Table A1:** Full item wording.

**Item**	**English wording used in the manuscript**
B1	I can use digital tools (e.g., PPT and teaching videos) to design physical education lesson plans.
B2	I can effectively use digital devices (e.g., projectors and tablet computers) to support teaching in physical education classes.
B3	I can adjust digital teaching content according to students’ physical fitness differences.
B4	I can assign physical activity tasks through digital platforms (e.g., WeChat or DingTalk) and track students’ completion.
B5	I can integrate wearable devices (e.g., sports bracelets) into teaching to monitor students’ physical performance and adjust teaching plans accordingly.
C1	I can search for and download digital resources suitable for physical education teaching from educational resource platforms.
C2	I can produce physical education teaching videos or animated demonstration content suitable for students.
C3	I can integrate multiple digital resources (e.g., videos, images, and data) to support physical education teaching.
C4	I can use digital tools (e.g., chart tools) to develop physical fitness test forms or exercise record cards.
C5	I can share my physical education teaching resources with other teachers through online platforms.
D1	I can use digital assessment tools (e.g., questionnaires and online tests) to understand students’ sports performance.
D2	I can use digital tools (e.g., Excel and data-analysis software) to analyze students’ physical fitness and sports achievement data.
D3	I can use visualization tools to display changes in students’ sports performance.
D4	I can provide students with personalized feedback on their exercise progress through digital platforms.
D5	I can adjust teaching plans or activity content based on students’ digital assessment data.
F1	I can interact with students through online discussion forums or social media tools and answer their physical education learning questions.
F2	I can guide students to use digital tools (e.g., exercise-recording apps) for independent exercise and learning.
F3	I can design digital tasks (e.g., step-count app goals) to motivate students to participate in physical activity.
F4	I can use digital teaching resources to improve students’ motor skills.
F5	I can stimulate students’ interest and enhance their engagement through online competitions or exercise challenge activities.
G1	I can proactively learn and master emerging digital teaching tools (e.g., VR fitness technologies).
G2	I can participate in professional development activities through digital platforms.
G3	I can use teaching reflection tools to document my teaching progress and make improvements.
G4	I can join educational communities to exchange experiences with peers.
G5	I can keep informed about and participate in national or regional digital education policies and programs.

The questionnaire was administered in Chinese. The English wording is provided for reporting purposes.
